# Utilization of labor pain management methods and associated factors among obstetric care givers at public health institutions of East Gojjam Zone, Amhara region, Ethiopia, 2020: a facility based cross – sectional study

**DOI:** 10.1186/s12884-022-05094-z

**Published:** 2022-11-01

**Authors:** Aster Shiferaw, Belsity Temesgen, Nakachew Mekonnen Alamirew, Tejitu Wube, Yichalem worku

**Affiliations:** College of medicine and health science, Department of Midwifery, Debre Markos University, Debre Markos, Ethiopia

**Keywords:** Labor pain management method, Utilization, Obstetric care giver, East Gojjam Zone

## Abstract

**Background:**

Labor Pain is “unpleasant sensory and emotional experience associated with actual or potential tissue damage, affects parturient and fetuses’. Developed countries regularly use obstetric analgesia but in developing countries, including Ethiopia pain is neglected and most women go through painful labor. The study was conducted in public health institutions of East Gojjam Zone; Amhara region, Ethiopia. The aim of this study was to assess utilization of labor pain management methods and associated factors among obstetric care givers in the study setting.

**Method:**

Facility-based cross sectional study design was carried out in public health institutions of East Gojjam Zone from April 15 to May 15, 2020. Semi Structured questionnaires were used and 305 obstetric care givers were participated. Stratified sampling technique was used. Data was entered by Epi- data version 3.1 and exported to SPSS version 20. Descriptive analysis was done and Bivariate and multivariate logistic regression with 95% CI was used to saw the association of dependent and independent variables at p < 0.05.

**Result:**

Utilization of labor pain management methods in this study was 48.9%. In Multivariate logistic regression; Professional knowledge [AOR = 2.006, 95% CI = ((1.032–3.898)], availability of drug and equipment [AOR = 2.937, 95% CI= (1.311–6.578)] and allow companionship [AOR = 2.587, 95% CI= (1.322–5.063)] were significantly associated with utilization of labor pain management methods.

**Conclusion and recommendation:**

This study showed low utilization of labor pain management methods. Adequate knowledge, allow accompany and availability of drug & equipment were factors associated with use of labor pain relief options .so it is important to build knowledge of obstetric care givers, availing drugs and materials and make safe the environment for accompany ship to improving use of labor pain management.

**Supplementary Information:**

The online version contains supplementary material available at 10.1186/s12884-022-05094-z.

## Background

Labor Pain is “an unpleasant sensory and emotional experience, which is a complex, subjective and multidimensional response that leads to actual or potential tissue damage [1, 2]. Also, it causes endorphin release which leads to three-to five-fold increment of plasma concentrations of adrenaline and noradrenaline [3]. It‘s advancement associated with ischemia of the uterus due to contraction, dilation of cervix, stretching of vagina, perineum and pelvic structures [2]. Even if it had no pathological process, most women stated labor pain is the most severe distressing event and painful experience. Due to that, most women are concerned about labor pain and how they can be relieved from pain [4–6].

Because labor pain is subjective and differs from woman to woman, so it is better to have an option according to their favorite and individual conditions [7]. Modern obstetrics set internalizing distress of the laboring mother and maintaining safe labor with minimum pain as one of the basic principles [8]. So awareness of labor pain helps to have stress on labor pain management with consideration of their choice [9]. Methods of labor pain relief should be effective, safe, woman-centered and not affect women’s mobility or the progress of labor [10, 11].

Pharmacological methods like pethidine (Meperidine) and other analgesics are used to effectively control labor pain [12, 13]. Additionally, herbal medicine and non-pharmacological methods like emotional support from partners ,family members, and professional or non- professional staff, assuming specific positions, moving, prayer for God to reduce their pain, breathing exercises, taking showers are used to control labor pain, which gives maternal birth satisfaction and good fetal outcome [13–20]. Then both pharmacological and non-pharmacological methods are important for relieving labor pain effectively [21].

Provision of pain relief in labor is neglected, without argument of the need, advantages and disadvantages of pain relief options, especially pharmacological options [22]. Lack of awareness, misunderstanding regarding acceptability, safety and availability of pain relief options and misconceptions like considering labor pain as normal and its interference is not good for health are considered to be the main reasons why women in many lower and middle income countries (LMIC) like Ethiopia, do not receive adequate pain relief [23, 24]. But, if labor pain exceeds a certain intensity and duration, it affects the hopeful mother and her attitudes towards her baby [25, 26].

Fear of severity of labor pain and lack of appropriate labor pain management for a laboring mother leads to concern about the approaching birth or requesting a caesarean section and exposes them to financial constraints [27]. About 36.4% of laboring mothers desired the procedure/caesarian section to avoid labor pain [28]. Also, women’s low satisfaction in intra partum care was associated with poor pain relief during labor and vaginal birth [29–32]. That influences a woman’s choice of place of delivery in Ethiopia [33]. Due to that, only 48% of births occur in health facilities from those receiving antenatal care [34]. In this case, utilization of labor pain management can be a great opportunity to enhance institutional delivery. Developed countries regularly use obstetric analgesia and stress on continuous labor support, but, in developing countries, including Ethiopia, pain is neglected, obstetrics services are poorly developed and most women still go through painful labor regardless of availability of methods [22, 35, 36]. Even if it is a serious component in the Ethiopian federal minister of health’s (EFMOH) efforts to advance the quality of maternal health services available to the Ethiopian public, its practice is not significantly accepted [37].

There are only a few published studies in Ethiopia on the magnitude of labor pain management methods and associated factors and in the study area one study was done only in health centers. All of those studies are conducted in the same level of health institution, either health centers or hospitals alone, which affects the scientific estimation and generalization for all levels of health institutions. Especially in hospitals where high level professionals are available, there is a strict follow-up to improve quality of care and respect maternal service rights, so the estimation may not be representative of other hospitals and health centers. So, to increase its representativeness, this study assessed the magnitude of use of labor pain management methods and associated factors by incorporating all levels of public health institutions (referral, general, primary hospitals and health centers) of the east Gojjam zone, Amhara, Ethiopia. The result of this study will be used as input for those who are interested in developing management guidelines on the use of labor pain relief options. Also, it helps to identify the gap and give consideration to improving quality of care. For academicians, it will help to give consideration to educating labor pain management options for their students in their pre-service and in-service setting. In addition, this study will be an input to further research activities by researchers. Also, it helps to update the magnitude and pattern of labor pain management methods for improvement.

## Methodology

### Study setting, design and period

Institutional based cross sectional study was conducted in public health institutions of East Gojjam Zone. East Gojjam is one of the administrative Zones in Amhara regional state of Ethiopia. It is bounded by the Oromia region in the south, on west by West Gojjam zone, on north by South Gondar zone, and on the east by South Wollo zone. Debre Markos is the capital city of East Gojjam Zone and located at 265 km from Bahir Dar and 299 km from Addis Ababa the capital city of the country. According to census of 2007, conducted by the central statistical agency of Ethiopia (CSA), the zone has a total population of 2,153,937, among this, 1,066,716 are men and 1,087,221 women; with an area of 14,004.47 square kilometers. East Gojjam zone has 19 woredas .It has one referral hospital, one general hospital, eight primary hospitals, and 102 health center. Currently there are 6 gynecologists, 107 general practitioners, 488 midwives, 22 integrated emergency surgical officers (IESO), 22 anesthetists, 841 nurses and 366 health officers providing maternal and child health (MCH) services in the above mentioned institutions. The study was carried out from April 15-May 15, 2020.

## Population

### Source population

All obstetric care givers who were working at labor ward in the public health institutions of East Gojjam Zone.

### Study population

Obstetric care givers who were working at labor ward in the selected public health institutions of East Gojjam Zone.

### Inclusion criteria

All obstetric care givers who were working at labor ward in the selected public health institutions of East Gojjam Zone.

## Sample size determination

### Sample size for outcome variable

The sample size was determined by using single population proportion formula.so.

$$n = \frac{{Z{\text{ }}{{\left( {\alpha /2} \right)}^2}*\left( p \right)*\left( {1 - q} \right)}}{{{d^2}}}$$

While n is sample size, Z (α/2) at 95% CI = 1.96, d is margin of error 5%=0.05 and p = 40.1% was the proportion of utilization of labor pain relief options of previous study in Amhara region [38] q = 1-p, 1-0.401 = 0.599 .

$$n = \frac{{{{\left( {1.96} \right)}^{2{\text{ }}*}}\left( {0.401} \right){\text{ }}*\left( {0.599} \right)}}{{{{\left( {0.05} \right)}^2}}} = 369$$

### Sample size by associated factor

The sample size was calculated by Epi Info version 7 by using associated factor of study done in Amhara region [38] and Tigray region [39] (Table 1).

**Table 1:** Sample size for second specific objective of utilization of labor pain management methods East Gojjam Zone, Amhara, Ethiopia 2020

Variable	Proportion	Power	CI	calculated sample size	Reference
Lower educational level	P1 = 29.03% P2 = 47.3%	80%	95%	242	[38]
Inadequate knowledge	P1 = 52.5% P2 = 32.6%	80%	95%	212	[38]
Positive attitude	P1 = 54.5 P2 = 28.7	80%	95%	128	[39]
High level of education	P1 = 66.7 P2 = 40.5	80%	95%	128	[39]

Because of the sample size calculated by outcome = 369 was greater than the calculated value by variable, so the sample size 369 was used to determine the total sample size. Because of that the source population (N = 1852) is less than 10,000 the sample size was adjusted by correction formula.

$$n = \frac{n}{{\frac{{1 + n}}{N}}} = 294$$

By adding 10% non-response rate the total sample size becomes 323.

### Sampling procedure

There are 112 health institutions in east Gojjam zone. So those were stratified as referral hospital, general hospital, primary hospital and health center .From each strata 25% of health institution which was 1 Referral hospital have 71 obstetric care givers, 1 General hospital have 28 obstetric care givers, 8 Primary hospitals have 32 obstetric care givers and 25 Health centers have 225 obstetric care givers were selected by simple random sampling technique with assumption of there is no variation of health institutions with in each strata. There were 356 obstetric care givers in the selected health institutions which are small in number to allocate proportionally. So only 323 self-administer questionnaires were prepared and provided per determined sample size as censes.

## Dependent variable and independent variable

### Dependent variable

Utilization of labor pain management methods.

### Independent variable

Socio demographic: age, sex, religion, qualification, profession, year of experience.

Individual factor: knowledge, attitude, pain expectation, care giver method preference,

Institutional factor: availability of analgesic agents and equipment’s, Allow companionship and Training.

### Operational definition

Utilization of Labor pain management method: respondents who answer greater than or equal to the mean value of use related labor pain management method questions.

Favorable attitude: respondents who answered greater than or equal to the mean value of attitude related labor pain management questions.

Unfavorable attitude: a respondent who answered less than to the mean value of attitude related labor pain relief method questions.

Adequate knowledge: respondents who knew greater than or equal to the mean values of knowledge related questions.

Inadequate knowledge: respondents who answered less than to the mean values of knowledge related labor pain relief method questions [39].

## Data collection tools and procedures

### Data collection tool

Semi-Structured and self-administered questionnaires were utilized after being constructed from different literatures and the data was collected from pre- tested questionnaires. The questionnaires consist of six portions; which were used to assess socio-demographic characteristics, knowledge and attitude of obstetric care givers, utilization, preference of labor relief methods and institutional factors affecting the use of labor pain management options [1–3]. The questionnaire was designed in English to be understandable and distributed to obstetric care givers at data collection time. Six BSc nurses and midwife teaching staff data collectors were recruited; a principal investigator and one MSc teaching staff member supervised the data collection.

### Data quality control

Training was provided to data collectors to for a common understanding of the study and the questioner. 5% of the sample pretest was conducted by a self-administered questioner to shape the questioner. There was frequent supervision during data collection; the questionnaire was reviewed and checked for completeness, accuracy and consistency by the principal investigator and supervisor. The code was given before the data entry.

### Data analysis

After data collection, data was entered into Epi-data version 3.1 and exported to Statistical Package for Social Science (SPSS) version 20 for further analysis. Descriptive statistics were used to determine frequencies and summary statistics. Primarily, bi-variable logistic regression was carried out to see the significant association of each of the independent variables with the outcome variables at 95% confidence interval and p-value less than 0.25. Then multivariable logistic regression was carried out for variables with a p-value less than 0.25 in bivariate logistic regression to determine significant relationships between the dependent and independent variables at p-value less than 0.05. Model fitness was checked by using Hosmer and Lemeshow’s goodness of fit. The result was presented in the form of text, tables and figures.

## Result

### Socio demographic characteristics of respondent

Three hundred five participants were responded to questioners with a response rate of 94.4% .The mean age of the respondents was 28.3 years (SD ± 4.1). The majority of the respondents, 213 (69.8%), were among the age group of 20–29 .Among the respondents, 195 (63.9%) were male and 287(94.1%) were orthodox religious followers. The majority of the respondents, 150 (49.2%), were midwives, 182 (59.7%) were BSc holders and 175 (57.4%) of respondents had less than 5 years’ experience (Table 2).

**Table 2:** Socio demographic characteristics of obstetric care giver who works labor ward in health institutions of east Gojjam zone, Amhara, 2020. (n = 305)

Characteristics	Frequency	Percent (%)
**Age group**		
20–29 yrs. 30–39 yrs. ≥ 40 yrs.	213 84 8	69.8 27.6 2.6
**Sex**		
Male female	195 110	63.9 36.1
**Religion**		
orthodox Muslim protestant	287 7 11	94.1 2.3 3.6
profession		
medical doctor midwife nurse health officer IESO anesthetist	12 150 60 61 8 14	3.9 49.2 19.7 20.0 2.6 4.6
Level of education		
gynecologist Resident doctor General practitioner MSc BSc Diploma	4 2 8 16 182 93	1.3 0.7 2.6 5.2 59.7 30.5
Clinical experience		
≤ 5 yrs. 6–9 yrs. ≥ 10 yrs.	175 93 37	57.4 30.5 12.1

### Knowledge and attitude of respondents

Among the total respondents, about 298(97.7%) knew about labor pain management methods. From them, 215 (70.5%) knew both pharmacological and non-pharmacological methods, 74(24.8%) only non- pharmacological and 9(3%) knew only pharmacological methods.

Of the pharmacological labor pain management methods, 126 (41.3%), 106 (34.8%), 95(31.1%) and 24 (7.9%) of respondents knew systemic opoid, regional analgesia, non-opoid systemic analgesia and inhalational respectively. And from non-pharmacological methods, the most well-known one is psychotherapy response by 219 (71.8%) respondents. Next 159 (52.1%), 155(50.8%), 150 (49.2%) and 127(41.6%) responds allow the mother to ambulate, massage the back ,relaxation & breathing technique and show the patient how to bear down respectively. The remaining were music therapy 62 (20. 3%), diversional therapy 22(7.2%), Transcutaneous electrical nerve stimulation 16(5.2%) and acupuncture 11 (3.6%).

In this study, among the total respondents, 126 (41.3%) had adequate knowledge from the listed knowledge related questions. The mean score of respondents was 4.4 with (SD ± 2.77).

From the respondents who knew about pharmacological methods, 167(54.8%) expect obstetric analgesia to have side effects on labor delivery outcome by causing 118 (38.7%) fetal distress, 111 (36.4%) in delay progress of labor, 62 (20.3%) increase in instrumental delivery and 7 (2.3%) other like bleeding .

Regarding respondents’ attitude towards labor pain management methods, 163 (53.4%) respondents had a favorable attitude. The mean score of respondents was 22.6 with (SD ± 4.1). More than half of the respondents, 181(59.3%), agree that labor pain management methods help laboring mothers to cope with labor pain. And also, 166(54.4%) agree that professionals have the responsibility and obligation of managing labor pain and 139(44.9%) agree that analgesia is necessary for managing labor pain.

### Personal Preference and Pain Expectation of Respondent

From the respondents, 161 (54%) prefer the non-pharmacologic method, 98 (32.9%) both pharmacologic& non-pharmacologic and 39(13.1%) the pharmacologic method to manage laboring mothers. The majority of respondents, 211 (69.2%) expect labor pain as severe pain, 71(23. 30%) and 23 (7.5%) expect moderate and mild respectively.

Institutional factor.

From respondents who knew pharmacological methods, 119 (71.3%), 100(59.9%) and 37 (22.2%) answered, as NSAID, petidine and epidural analgesia respectively were available in their health institution. And 163 (53.4%) of the respondents reported their health institution allowing a companion for choice of laboring mother. From the total, respondents only 33(10.8%) had got training regarding managing labor pain.

### Utilization of labor pain management methods

**Fig. 1:**
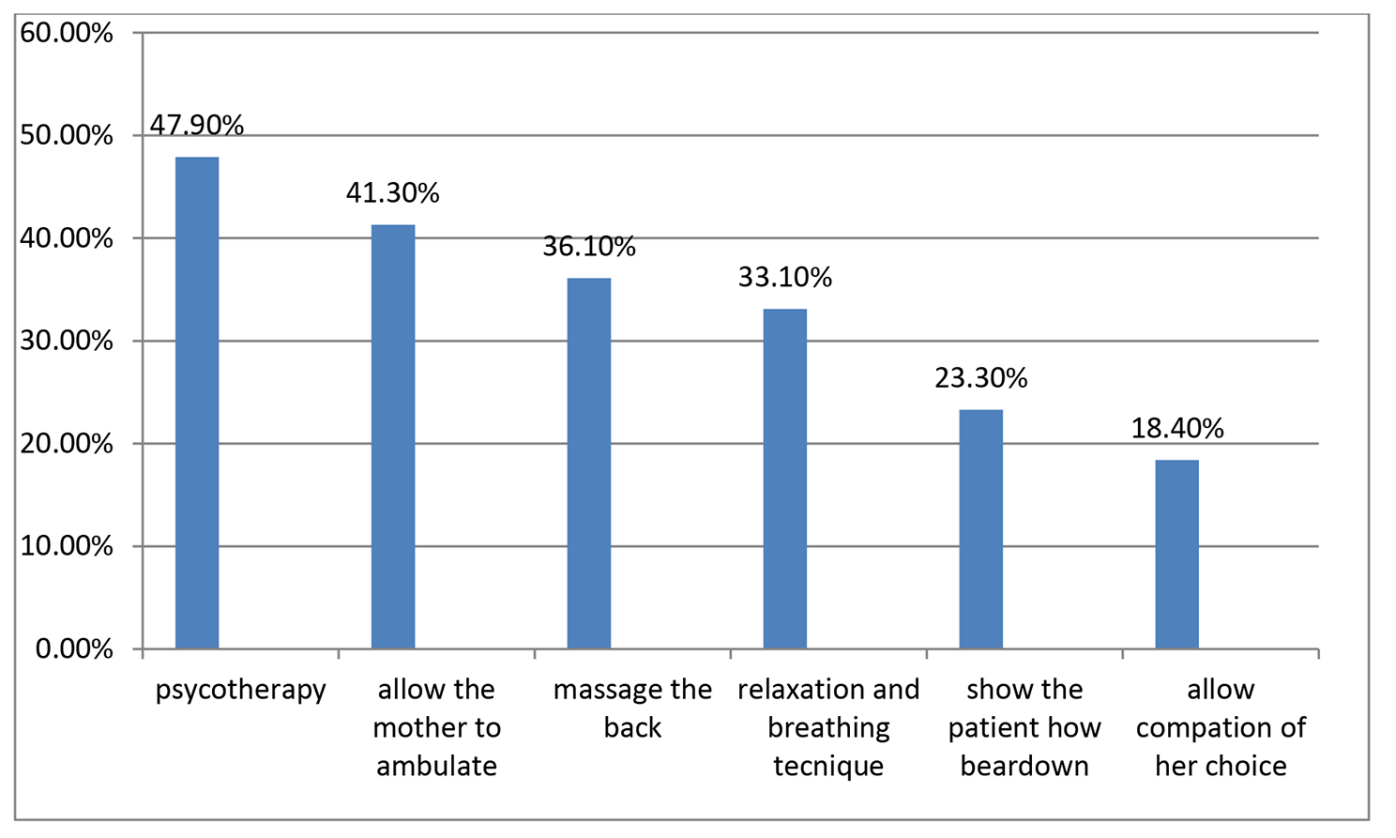
A bar chart shows the utilization of non-pharmacological methods among obstetric care givers in public health institutions of east Gojjam zone, Amhara 2020

The overall utilization of labor pain management methods in this study was 48.9% with a 95% confidence interval [44.3–54.1] and the mean score of utilization was 2.7 (SD ± 2.4). Among these, 41.3% contribute to non-pharmacologic methods and 4.6% to pharmacologic methods. The most widely used non-pharmacological method was 146(47.9%) psychotherapy followed by 126(41.3%) allowing the mother to ambulate and 110 (36.1%) massage the back (Fig. 1). Also from pharmacological labor pain management methods 62 (20.3%) of respondents used paracetamol, 60 (19.7%) petidine, and 54(17.7%) non-steroidal anti-inflammatory drug (NSAID). The majority, obstetric care givers, 152 (67.6%) use labor pain management methods sometimes, 52 (23.1%) use them routinely and 21 (9. 3%) use them on client request.

### Associated factor of utilization of labor pain management methods

Based on bi-variable analysis, obstetric care giver experience, knowledge, availability of analgesia, attitude, profession, age, allowing companion for laboring mother and training were associated with utilization of labor pain management methods at p - value less than 0.25 and a candidate for multi-variable logistic regression. In multi variable analysis knowledge, allowing companies and availability of analgesia were significantly associated with utilization of labor pain management methods at p - value less than 0.05.

Obstetric care givers who had adequate knowledge were 2.01 times more likely to utilize labor pain management methods than those who had inadequate knowledge, [AOR = 2.006, 95% CI = (1.032–3.898)] .Obstetric care givers, in the institution which allow companion for choice of laboring mothers were 2.59 times more likely to utilize labor pain management methods than those in the institution not allow companion, [AOR = 2.587, 95% CI= (1.322–5.063)]. Also, obstetric care givers, in institutions which had availability of analgesia drugs were 2.94 times more likely to utilize labor pain management methods than those in the institution who had no availability of analgesia drugs.[ AOR = 2.937, 95% CI= (1.311–6.578) ] or when drugs were not unavailability in the institutions, obstetric care givers were 68% times less likely to utilize labor pain methods than when drugs were available [AOR = 0.317,95% CI= (0.167–0.601) Table 3.

**Table 3:** Multivariate logistic analysis of associated factors of utilization of labor pain management methods among obstetric care giver in health institutions of East Gojjam Zone, Amhara 2020

Variables	Utilization of labor pain management methods		COR(95% CI)	AOR(95% CI)			P value		
	Yes	No							
**Age group**									
20–29 ≥ 30	163 62	50 30	1.577(0.920-2.704) 1.00			1.263(0.507-3.147) 1.00	0.616		
Experience									
<=5 6–9 > 10	130 73 22	45 20 15	1.970(0.941-4.123) 2.489(1.094–5.661) 1.00			1.494(0.452-4.938) 2.985(0.942-9.462) 1.00	0.510 0.063		
Profession									
Midwife Other	117 108	33 47	1.543(0.921- 2.586) 1.00			0.970(0.482-1.951) 1.00	0.931		
**Knowledge**									
Adequate Inadequate	102 123	24 56	1.935(1.121–3.339) 1.00			**2.006 (1.032–3.898)** 1.00	**0.04**		
**Companion**									
Yes No	135 90	28 52	2.786(1.638–4.739) 1.00			**2.587 (1.322–5.063)** 1.00			**0.006**
**Availability**									
No Yes I don’t know	18 132 14	18 34 8	1.00 3.882(1.826–8.254) 1.750(0.590-5.189)			1.00 **2.937 (1.311–6.578)** 2.276 (0.710-7.296)			**0.009** 0.167
Training									
No Yes	197 28	75 5	1.00 2.132(0.794-5.726)			1.00 1.287 (0.403-4.112)		0.671	
Attitude									
Favorable attitude unfavorable attitude	127 98	36 44	1.584(0.948-2.646) 1.00		1.456 (0.755-2.808) 1.00			0.262	

Others: medical doctor, nurse, integrated emergency surgical officers (IESO), anesthetist and health officer

## Discussion

The overall utilization of labor pain management methods among obstetric care givers was 48.9% and 41.3%, contributing to non-pharmacological methods.

The result of this study is in line with a study conducted in Zaria, Nigeria in which 48.4% of respondents provided pain relief during labor, the most commonly used method was systemic opioids, but in this study the most commonly used one was psychotherapy [36]. The difference may be due to different awareness of pharmacological methods and differences in socio-demographic characteristics. In this study, use of non-pharmacological methods was 41.3%, which is in line with studies conducted in Egypt, which was 44.9% [10]. In this study, Psychotherapy was the most used non-pharmacological method. The finding is similar to a study conducted in Moshi, Tanzania. Most health care providers offer non-pharmacological methods like counseling about severity and nature of labor, psychological support and reassurance [40]. In this study, the use of pharmacological methods was 4.6%. This result differs from a study conducted in Colombia on unequal distribution of epidural analgesia in developed versus developing countries, which shows 75% in France, 71 in Sweden and 31.5% in Colombia [41]. This variation may be due to differences in socioeconomic status, a great awareness of laboring mothers and professionals about pharmacological methods and availability of drugs & materials in this developed country.

The result in this study is greater than studies conducted in Kembata Tembaro Zone, Southern Ethiopia 37.9% [42] Amhara region referral hospitals 40.1% [1] and Tigray region general hospitals 43.3% [39] on labor pain management methods. This difference may be due to changes in the study area; this study incorporates all levels of education of obstetric care givers & health institutions and variation of sample size. The result of this study is less than the study done in Addis Ababa which was 54.2% [43]. This may be due to socioeconomic differences, advancement of the institution to give attention to labor pain and maternal awareness of labor pain management options.

In current study knowledge, allowing companionship and availability of drugs and equipment were significantly associated with utilization of labor pain management methods. In this finding, adequate knowledge of the obstetric care giver is the higher odds of utilization of labor pain management methods. This result is inconsistent with the study conducted in Amhara region referral hospitals [38] and a study conducted in Kembata Tembaro Zone, Southern Ethiopia, obstetric care givers who had inadequate knowledge were more likely to use labor pain management methods than those who had adequate knowledge [42]. The difference may be due to this study incorporating higher level professionals and all levels of health institutions.

Allowing companions for the choice of a laboring mother is significantly associated with utilization of labor pain management methods. Obstetric care givers in institutions allow companions higher the odds of utilization of labor pain management methods. Obstetric care givers who were in the institution which allow companions to choose of laboring mother were 2. 59 times more likely to utilize labor pain management methods than those who were in the institution not allow companions [AOR = 2.587, 95% CI= (1.322–5.063)].

Also, availability of drugs and materials was significantly associated with the utilization of labor pain management methods. That is because institutional availability of drugs and equipment is the higher the utilization of labor pain management methods. Obstetric care givers were 2.94 times more likely to utilize labor pain management methods when analgesia drugs are available in the institution than analgesia drugs not available. This result is in line with a study conducted in Kembata Tembaro Zone ,Southern Ethiopia [42] and Addis Ababa public hospitals ,obstetric care givers were less likely to utilize labor pain management methods when drugs and materials were not available [43].

## Conclusion and recommendations

However, relieving the pain of the laboring mother has a great initiating role in the consumption of obstetric care providing services; in developing country including Ethiopia utilization of labor pain management methods and incorporating it in care delivery system is not a consideration .This study shows that utilization of labor pain management methods was low. Professional knowledge, allowing companies for maternal choice and availability of drugs and equipment were significantly associated with the utilization of labor pain management methods. So preparing standardizes management protocol based on research and maternal exit interview, preparing an experience sharing program and onsite training for professionals to build and update their knowledge, Modifying their institution in a way that enables them to manage a laboring mother in a partition with their choice of company to increase their satisfaction and make avail necessary drugs and equipment is required for proper utilization of labor pain management methods.

## Electronic supplementary material

Below is the link to the electronic supplementary material.

**Supplementary Material 1**

## Data Availability

The data and materials that used to generate this manuscript and the manuscript itself can be accessed from the corresponding author through an email astershiferaw21@gmail.com.

## Abbreviations

AOR: Adjusted Odds Ratio
CI: Confidence Interval
COR: Crude Odd Ratio
CSA: Central Statistical Agency of Ethiopia
IESO: Integrated Emergency Surgical Officers
LMIC: lower and middle income country
MCH: Maternal and Child Health
SPSS: Statistical Package for Social Sciences
