# Supplementary material for: Utilization of labor pain management methods and associated factors among obstetric care givers at public health institutions of East Gojjam Zone, Amhara region, Ethiopia, 2020: a facility based cross – sectional study
Source: BMC Pregnancy Childbirth. 2022 Nov 1;22:803. doi: 10.1186/s12884-022-05094-z (PMC9623903; doi:10.1186/s12884-022-05094-z)
Supplement: Supplementary file 1 — Supplementary Material 1 [file 12884_2022_5094_MOESM1_ESM.docx]

# ANNEXES

**Annexes I: English Version Information Sheet**

Questionnaire Identification Number ____________

Dear Respondent:

Good morning/Afternoon! My name is __________________________. I am a data collector in the research conduct by Aster Shiferaw on utilization of labor pain management and associated factors among obstetric care giver in East Gojjam Zone public health institutions. You are selected and included in the study as part of the sample population to complete the questionnaire considered by the researcher. Your honest response is vital to assess the utilization of labor pain management and associated factors of obstetric care giver

**Name of the organization**: Debre Markos University, College of Health Sciences, department of Midwifery.

**Name of the Sponsor**: Debre Markos University

**Introduction**: Information sheet and consent form prepare for obstetric care giver who provide obstetric care in hospitals of East Gojjam zone. Individuals, who available during data collection will participate in a research project, which is conduct to assess utilization of labor pain management and associated factors of obstetric care giver of East Gojjam Zone

**Purpose of the study:** The aim of this study is to assess utilization of labor pain management and associated factors of obstetric care giver of East Gojjam Zone public health institutions. Assessing of utilization of labor pain relief methods and associated factors of obstetric care giver is important to enhance the quality of obstetric care especially labor pain management so as to increase maternal satisfaction which may contribute for utilization of institutional delivery. The results of the study will be used to evaluate and develop appropriate strategies to address the problems related to labor pain management utilization and associated factor in obstetric care giver of East Gojjam Zone public health institutions.

**Procedure:** To assess utilization of labor pain relief methods and associated factors of in obstetric care giver of East Gojjam Zone public health institutions you are invited to participate in this project. If you are volunteer to participate, I need you to clearly understand the aim of this study and show your agreement. Finally, you are kindly requested to give your frank response to the questionnaire. All your responses and the results obtained will keep confidential where by no one could have access to your response.

**Benefits / Risk/ Discomfort:** When you participate in this research project you may feel some discomfort by wasting your time (15-25mintues). However, your participation is definitely important to assess utilization of labor pain relief methods and associated factors of obstetric care giver to develop appropriate strategy & to improve maternal health services in public health institutions of East Gojjam Zone. I am sure there will no risk in participating in this research project. You will not provide any incentive or payment to take part in this study.

**Confidentiality:** The information collected from you will keep strictly confidential and stored in a file, without your name by assigning a code number to it. The information obtained in this study will used only for research purposes.

**Right to refuse or withdraw**: You have the full right to refuse from participating in this research. You have also the full right to withdraw from this study at any time you wish. You can choose not to respond to some or all questions if you do not want to give your response.

**Annex II: English Version Consent Form**

The selected participant should have read and understood the information sheet carefully. I understood the purpose, the benefit of the study and personal information regarding me and all answers given by me should not be transferred to the third party without my permission. I also understood that I can decide whether or not to take part in the study or even take out from the study at any time so that I agree to participate in the study with my signature below.

The participant Sign_______________________

Supervisor Name _____________________________ signature_________ Date_____/_______/____E.C.

Time Started: Hour: _____ Minute: _____

Questionnaire No____________________________

Time ended: Hour: _____ Minute: _____

Name of Data collector **__________________________________________________**

Date **______/_____/____ ____**E.C**.** signature**___________**

**Annex III: English questioner**

Circle or write the appropriate response

| 1. **Background characteristics of the respondent** | | | | | | | | | | |
| --- | --- | --- | --- | --- | --- | --- | --- | --- | --- | --- |
| S.no | | Question | Response | Skip | | | | code | | |
| 01 | | Age in year? | _______________ |  | | | |  | | |
| 02 | | Sex | 1. Male 2. Female |  | | | |  | | |
| 03 | | Religion | 1. Orthodox 2. Muslim 3. Protestant 4. Other…… |  | | | |  | | |
| 04 | | Profession | 1. Medical doctor 2. Midwife 3. IESO 4. Anesthetics |  | | | |  | | |
| 05 | | Qualification | 1. Gynecologist 2. Resident doctor 3. General practitioner 4. MSc 5. BSc 6. Diploma |  | | | |  | | |
| 06 | | Clinical experience in year? | ____________________ |  | | | |  | | |
| 1. **Knowledge related questions** | | | | | | | | | | |
| 07 | Do you know about labor pain management options? | | 1. Yes 2. No | | If “no” for 08 skip to | | | | 13 | |
| 08 | If yes for no 07 what type of labor pain management do you know? More than one answer is possible | | 1. Pharmacologic 2. Non pharmacologic 3. Both | |  | | | |  | |
| 09 | If “A&C” to question No 8, which pharmacologic method do you know? More than one answer is possible | | 1. Regional analgesia 2. Systemic opioids 3. Inhalational 4. Non opoid systemic analgesia 5. If other specify ----- | |  | | | |  | |
| 10 | If “B &C” to question No 8, which type of non-pharmacologic method, do you know? More than one answer is possible | | 1. Psychotherapy 2. Show the patient 3. How to bear down 4. Massage the back 5. Trans cutaneous electrical nerve stimulation 6. Diversional therapy 7. Acupuncture 8. Allow companion of her choice 9. Relaxation/breathing technique 10. Allow the mother to ambulate 11. Subcutaneous water injection 12. Hypnosis 13. Music therapy 14. Other specify ---------------- | |  | | | |  | |
| 11 | If you know about labor analgesia, do obstetrics analgesia have side effect on labor and delivery outcome? | | 1. Yes 2. No 3. I don’t know | | If B &C for 12 skip to | | | | 14 | |
| 12 | If” yes” to question 11, what is the side effect of analgesia on labor and its outcome? More than one answer is possible | | 1. Delay progress of labor 2. Cause fetal distress 3. Increase instrumental delivery 4. If other specify ------ | |  | | | |  | |
| 1. **Attitude related questions** | | | | | | | | | | |
| 13 | Administering labor pain analgesics for mothers during labor is important for better outcome of the delivery for both the mother and the neonate | | 1. Strongly Disagree 2. Disagree 3. Neutral 4. Agree 5. Strongly agree | | |  | | |  | |
| 14 | Labor pain management methods can help the mother to cope labor pain? | | 1. Strongly Disagree 2. Disagree 3. Neutral 4. Agree 5. Strongly agree | | |  | | |  | |
| 15 | In managing of labor pain, stage of labor has no object | | 1. Strongly Disagree 2. Disagree 3. Neutral 4. Agree 5. Strongly agree | | |  | | |  | |
| 16 | Every mother pain during labor should be managed | | 1. Strongly Disagree 2. Disagree 3. Neutral 4. Agree 5. Strongly agree | | |  | | |  | |
| 17 | Labor pain is natural and mother hasn’t to face it | | 1. Strongly Disagree 2. Disagree 3. Neutral 4. Agree 5. Strongly agree | | |  | | |  | |
| 18 | Professionals has responsibility and obligation of managing of labor pain | | 1. Strongly Disagree 2. Disagree 3. Neutral 4. Agree 5. Strongly agree | | |  | | |  | |
| 19 | Analgesic is necessary for managing labor pain | | 1. Strongly Disagree 2. Disagree 3. Neutral 4. Agree 5. Strongly agree | | |  | | |  | |
| 1. **Utilization related questions** | | | | | | | | | | |
| 20 | Did you utilize any form of labor pain management methods currently | | 1. Yes 2. No | | | If “B” for 21 skip to | | | | 27 |
| 21 | If” yes” to question No 20, what is your pattern of utilization? | | 1. Routinely 2. Sometimes 3. on patient request | | |  | | | |  |
| 22 | If” yes” to question No 20 which method? You can answer more than one | | 1. Pharmacologic 2. non-pharmacologic 3. Both | | | If “B” for 23 skip to | | | | 26 |
| 23 | If “A&C” for question No 22, which type? You can answer more than one | | 1. Pethidine 2. Paracetamol 3. Epidural analgesia 4. NSAID 5. Inhalational 6. If other specify -------- --------- | | |  | | | |  |
| 24 | If “B&C” for question No 22, which type? You can answer more than one | | 1. Psychotherapy 2. Massage the back 3. Allow companion of her choice 4. Relaxation/ breathing technique 5. Acupuncture 6. Show the patient how to bear down 7. Hot or cold pack compress 8. Hypnosis 9. Allow mother to ambulate/labor exercise 10. Subcutaneous water injection 11. If other specify ---------------- | | |  | | | |  |
| 1. **Question related to individual factor** | | | | | | | | | | |
| 25 | If you know labor pain management methods, which method you prefer for managing labor pain? | | 1. Pharmacologic 2. Non-pharmacologic 3. both | | | |  | | |  |
| 26 | What is your expectation of labor pain? | | 1. Mild pain 2. Moderate pain 3. sever pain | | | |  | | |  |
| 27 | Have you attend education parallel to your work | | 1. Yes 2. No | | | |  | | |  |
| 1. **Questions related to institutional factors** | | | | | | | | | | |
| 28 | If you know pharmacologic methods, is their labor pain managing drugs/ analgesics available at your hospital? | | 1. Yes 2. No 3. I don’t know | | | |  | | |  |
| 29 | If yes to question No 28, which type? You can answer more than one | | 1. Pethidine 2. NSAID 3. Drug for epidural analgesia 4. If other specify ----------- | | | |  | | |  |
| 30 | Have you got any special training for managing labor pain? | | 1. Yes 2. No | | | |  | | |  |
| 31 | Does your hospital allow companion for choice of laboring mother? | | 1. Yes 2. No | | | |  | | |  |
